# Two Newly Isolated *Enterobacter*-Specific Bacteriophages: Biological Properties and Stability Studies

**DOI:** 10.3390/v14071518

**Published:** 2022-07-12

**Authors:** Martyna Cieślik, Marek Harhala, Filip Orwat, Krystyna Dąbrowska, Andrzej Górski, Ewa Jończyk-Matysiak

**Affiliations:** 1 Bacteriophage Laboratory, Department of Phage Therapy, Hirszfeld Institute of Immunology and Experimental Therapy, Polish Academy of Sciences, 53-114 Wrocław, Poland; martyna.cieslik@uj.edu.pl (M.C.); filip.orwat@hirszfeld.pl (F.O.); andrzej.gorski@hirszfeld.pl (A.G.); 2 Laboratory of Phage Molecular Biology, Department of Phage Therapy, Hirszfeld Institute of Immunology and Experimental Therapy, Polish Academy of Sciences, 53-114 Wrocław, Poland; marek.harhala@hirszfeld.pl (M.H.); krystyna.dabrowska@hirszfeld.pl (K.D.); 3 Phage Therapy Unit, Hirszfeld Institute of Immunology and Experimental Therapy, Polish Academy of Sciences, 53-114 Wrocław, Poland; 4 Department of Clinical Immunology, Infant Jesus Hospital, The Medical University of Warsaw, 02-006 Warsaw, Poland

**Keywords:** bacteriophages, *Tevenvirinae* subfamily, myoviruses, *Enterobacter cloacae*, antibiotic resistance, phage stability, urinary tract infection

## Abstract

In an era of antibiotic therapy crisis caused by spreading antimicrobial resistance, and when recurrent urinary tract infections constitute a serious social and medical problem, the isolation and complex characterization of phages with a potential therapeutic application represents a promising solution. It is an inevitable, and even a necessary direction in the development of current phage research. In this paper, we present two newly isolated myoviruses that show lytic activity against multidrug-resistant clinical isolates of *Enterobacter* spp. (*E. cloacae*, *E. hormaechei*, and *E. kobei*), the genomes of which belong to a poorly represented phage group. Both phages were classified as part of the *Tevenvirinae* subfamily (Entb_43 was recognized as *Karamvirus* and Entb_45 as *Kanagawavirus*). Phage lytic spectra ranging from 40 to 60% were obtained. The most effective phage-to-bacteria ratios (MOI = 0.01 and MOI = 0.001) for both the phage amplification and their lytic activity against planktonic bacteria were also estimated. Complete adsorption to host cells were obtained after about 20 min for Entb_43 and 10 min for Entb_45. The phage lysates retained their initial titers even during six months of storage at both −70 °C and 4 °C, whereas storage at 37 °C caused a complete loss in their activity. We showed that phages retained their activity after incubation with solutions of silver and copper nanoparticles, which may indicate possible synergistic antibacterial activity. Moreover, a significant reduction in phage titers was observed after incubation with a disinfectant containing octenidinum dihydrochloridum and phenoxyethanol, as well as with 70% ethanol. The observed maintenance of phage activity during incubation in a urine sample, along with other described properties, may suggest a therapeutic potential of phages at the infection site after intravesical administration.

## 1. Introduction

Bacteria of the *Enterobacter* species were first described in 1960 by Hormaeche and Edwards as Gram-negative, facultative anaerobic bacilli with the ability to move [1]. They belong to the ESKAPE (*Enterococcus faecium*, *Staphylococcus aureus*, *Klebsiella pneumoniae*, *Acinetobacter baumannii*, *Pseudomonas aeruginosa*, and *Enterobacter* spp.) group of pathogens, which include bacteria that are characterized by a constantly increasing resistance to antibiotics—this is a huge problem, particularly for hospital environments [2,3]. The name of this group of microorganisms is related to their “escape” from the effects of antibiotics and other antimicrobial agents [4,5]. According to a report by the World Health Organization (WHO) published in 2017 on the need to search for new antibiotics and ways to combat multidrug-resistant pathogens, bacteria from the *Enterobacteriaceae* family (including *Enterobacter* spp.) belong to the highest priority group [6]. Infections with these pathogens are especially dangerous in immunocompromised patients, e.g., in solid organ transplant recipients on chronic immunosuppressive therapy [7] or in hemodialysis patients [8]. *Enterobacter cloacae*, the main representative of *Enterobacter* spp., colonizes the mucosa of the human intestine in a physiological state; however, it is also described as the source of many different disorders, including bacteremia [9,10], urinary tract infections (UTIs) [11,12], wound infections [13,14,15], osteoarticular infections [16,17,18], and pneumonia/lower respiratory tract infections [19,20,21]. These bacteria also commonly colonize in various hospital wards, including neonatal intensive care units, which may result in the development of life-threatening infections such as meningitidis [22]. Moreover, it has been suggested that the presence of *E. cloacae* in the colon environment may be associated with the initiation and progression of colorectal cancer [23].

A significant proportion of clinical bacterial isolates are extended-spectrum β-lactamase (ESBL)-producing strains. The principal described genes responsible for this mechanism of resistance to β-lactam antibiotics (penicillins, cephalosporins, and monobactams) include *bla*_TEM_, *bla*_CTX-M_, and *bla*_SHV_ [20,24,25]. Broad-spectrum antibiotic resistance also includes resistance to carbapenems, known as last-resort antibiotics, and to colistin, which often causes a wide range of side effects [26,27]. Related genes that have increasingly been found in clinical strains are, among others, *bla*_KPC−2_, as well as *bla*_NDM_, and the presence of efflux pumps is also important [25,27,28]. Recent studies describing *Enterobacter*-induced UTIs indicate the involvement of extensive drug resistant (XDR), multidrug resistant (MDR), as well as carbapenem-resistant *Enterobacterales* (CRE) strains in the pathogenesis of infection in hospitalized patients in Eastern Europe [29].

In an era of increasing antibiotic resistance, the search for new alternative infection treatment strategies is a major medical challenge. Although antibiotics are losing their effectiveness, one way to combat bacterial pathogens is bacteriophage therapy. Bacteriophages (phages)—viruses specific to their bacterial host—after penetrating into the host pathogen, amplify and destroy them, and should not negatively impact the physiological microbiota [30]. Various environmental samples are used to search and isolate new therapeutic bacteriophages [31]. Despite the ubiquitous presence of phages in the environment, there may be many obstacles in their isolation, as well as the possibility of using them as therapeutic agents. There is a constant need to obtain preclinical data on new phages in order to assess their efficacy and safety, both in vitro and in vivo [32], especially in the case of urinary tract infections in which *Enterobacter* strains are etiological agents. In our study, we have isolated and characterized two new bacteriophages which are specific for multidrug-resistant (MDR) *Enterobacter* spp. strains, and which are meant to be applicable in phage therapy.

## 2. Materials and Methods

### 2.1. Bacterial Strains

In the study, we used twenty clinical strains of antibiotic-resistant *Enterobacter*, which were isolated from patients with infections of the genitourinary system (*n* = 7); ears (*n* = 2); and wounds, ulcers, or purulent lesions (*n* = 11); who applied to qualify for experimental bacteriophage therapy at the Ludwik Hirszfeld Institute of Immunology and Experimental Therapy, Polish Academy of Sciences (HIIET PAS) in the years 2017–2021. The species of bacterial strains were determined using matrix-assisted laser desorption/ionization—time of flight (MALDI-TOF/TOF) mass spectrometry (Bruker Daltonics, Billerica, MA, USA). Antibiotic sensitivity was determined by the microdilution method using the MicroScan WalkAway analyzer (Beckman Coulter, Brea, CA, USA). All bacteria were grown on MacConkey agar (incubation at 37 °C, approximately 18 h), but were stored frozen in a 20% glycerol (Difco) solution at −70 °C prior to the experiments.

### 2.2. Bacteriophage Isolation and Amplification

Almost three hundred and forty environmental samples (such as water samples from hospital and municipal wastewater, rivers, lakes, seas, fountains, households, and yards) from various geographical locations, from the collection of HIIET, were used to isolate new bacteriophages against *Enterobacter* spp. Water samples were stored as raw, incubated (i.e., mixed with peptone water and incubated at 37 °C before filtering), or 20 times concentrated (by tangential filtration with the use of a Vivaflow 200 ultrafiltration kit with a Hydrosart membrane and a MWCO 30 kDa operating at a pressure of 3 bar). A phage search based on the method described by Ślopek et al. (1983) [33] was used. For this purpose, bacterial suspensions were made in sugar broth (in the composition: meat extract 0.4 g, enzymatic hydrolysate of casein 5.4 g, yeast hydrolysate 1.7 g, Bacto Peptone 4.0 g, NaCl 3.5 g, glucose 10 g—per 1000 mL of H_2_O) and incubated for 2 h at 37 °C to obtain an optical density at wavelength λ = 600 nm (OD_600_) 0.4–0.6 (bacteria in logarithmic (midlog) phase growth), measured spectrophotometrically (BioSpectrometer basic, Eppendorf, Hamburg, Germany). The bacterial suspensions were subsequently poured onto plates with solidified agar intended for the cultivation of Gram-negative bacteria (pH = 7.4; composition in % *w*/*v*: distilled water, 1.2% agar (bioMerieux, Durham, NC, USA), 1.0% tryptone—enzymatic casein hydrolysate (bioMerieux, Durham, NC, USA), 0.5% NaCl (POCH, Gliwice, Poland), 0.3% meat extract (bioMerieux, Durham, NC, USA), 0.3% Na_2_HPO_4_ (POCH, Gliwice, Poland)), dried for a few minutes at room temperature (RT) and then for 1 h at 37 °C. Then, each environmental water sample was spotted on the plates, which were incubated for 3.5 h at 37 °C. Plates were left at 4 °C overnight and the results were read the next day. Complete/semicomplete lysis of the bacteria or single plaques at the site where the sample was spotted were considered a positive result. To isolate the phage, 200 µL of a two-hour culture of the host bacteria was suspended in 10 mL of peptone water (in the composition: meat extract 0.4 g, enzymatic hydrolyzate of casein 5.4 g, yeast hydrolyzate 1.7 g, Bacto Peptone 4.0 g, NaCl 3.5 g—per 1000 mL of H_2_O), and then 200 µL of the specific water sample was added, vortexed, and placed in an incubator (37 °C) overnight. The samples were filtered using syringe bacteriological filters with a pore diameter of 0.22 µm (Millipore, Burlington, MA, USA). Their serial dilutions (10^0^–10^−8^) were completed and 50 µL of each were spotted on the previously prepared plates with bacterial lawn (routine test dilution—RTD method). After incubation (3.5 h at 37 °C and then overnight at 4 °C), the number of plaques was counted. Phage dilutions, in which the 10–40 plaques were present, were then used in the double-layer agar method [34,35,36,37]. Briefly, 100 µL of the host bacteria and 200 µL of the given phage lysate’s dilution were added to 2 mL of melted 0.7% agar, mixed, and poured onto plates with already solidified agar. Plates were incubated for 4 h at 37 °C, then at 4 °C, and the phage titers were calculated the next day.

### 2.3. Assessment of the Lytic Spectrum of Phages

For the evaluation of the lytic spectrum of each phage, the bacterial suspensions of each of 20 *Enterobacter* spp. strains were used, with the subsequent procedure being very similar to that used in the search for new bacteriophages. Each amplified phage lysate (40 µL), at a titer of approximately 10^7^–10^8^ PFU/mL, was spotted on the plates with a bacterial lawn, incubated under the same conditions, and assessed the next day according to the signs: CL—confluent lysis, SCL—semiconfluent lysis, +++—60 and more confluent plaques, ++—20–60 separate plaques, +—10–20 separate plaques, +/−—single plaques, OL—opaque lysis. The percentage of bacterial strains that were sensitive to specific phages made it possible to determine their lytic spectrum.

In order to investigate the specificity of the lytic activity of bacteriophages, phage typing was also performed on strains of other species from the ESKAPE group: *E. faecalis* (*n* = 2), *S. aureus* (*n* = 2), *K. pneumoniae* (*n* = 1), *A. baumannii* (*n* = 3), *P. aeruginosa* (*n* = 2), and *Escherichia coli* (*n* = 2).

### 2.4. Assessment of Optimal Multiplicity of Infection (MOI) for Phages

During the experiments, the best MOI was assessed to optimize bacteriophage amplification. In the first stage, plots of dependence of bacterial concentration (colony-forming unit per milliliter—CFU/mL) on the OD_600_ that were determined spectrophotometrically were made for bacterial hosts. Briefly, different dilutions (0, 2, 3, 5, 8, and 10-fold) of the bacterial suspension were prepared and the OD_600_ of each sample was measured. Then, serial dilutions of each sample (from 10^0^ to 10^−8^) were made and 100 µL of the selected samples was spotted on an agar plate and spread over the entire surface. Plates were incubated at 37 °C for 11 h, then bacterial colonies were counted and bacterial titers that depend on optical density (OD_600_) were determined. In the next step, two-hour bacterial suspensions were made, and the bacterial concentrations were tested using the formulas generated from the plots (dependence of the bacterial titer in CFU/mL on OD_600_). Dilutions were then made to obtain a titer of about 3.0 × 10^6^ CFU/mL. Different titers of phage lysates were also prepared. To 10 mL of peptone water, we added 200 µL of a suspension of the appropriate bacterial strain and 200 µL of a specific phage dilution (10^3^, 10^4^, 10^5^, 10^6^, 10^7^, 10^8^, or 10^9^ PFU/mL) and mixed them. After overnight incubation at 37 °C, all samples were filtered and RTD in triplicate were performed for each of them to determine phage titers.

### 2.5. Planktonic Bacterial Lysis Assay

The experiment was performed similarly to those described elsewhere [38]. The lytic efficiency of the two new bacteriophages was determined using suspensions with different phage and bacteria ratios (at the most effective MOI determined in the previous experiment: 0.001, 0.01, 0.1, and 1.0). Briefly, the two-hour bacterial suspension was diluted to a titer of 10^6^ CFU/mL and samples with different MOIs were prepared. In these samples, as well as the samples with bacterial suspension (positive control) and only phage lysates (negative control), the OD_600_ were measured spectrophotometrically at 0, 1, 2, 3, 4, 6, 8, and 10 h of incubation at 37 °C. Two independent experiments were conducted, and in each of them, sample measurements were performed in triplicate.

### 2.6. Determination of the Phage Adsorption Time

To determine the kinetics of phage adsorption to their hosts and to estimate the adsorption rate constant, we used the procedures described elsewhere [39,40], with some modifications. Briefly, 10 mL of phage lysate (10^7^ PFU/mL) was added to 10 mL of bacterial suspension (10^8^ CFU/mL) at MOI = 0.1, and then incubated at 37 °C. At time 0 and after specified time intervals, 1 mL of the sample was taken and filtered through a syringe filter. Its serial dilutions were made and the titer of free, unadsorbed phages in the solution was determined. The number of phage particles at time 0 (immediately after mixing bacteria and viruses) represented 100% of the unadsorbed phages. Two independent experiments were conducted, and in each of them, sample measurements were performed in triplicate. The adsorption rate constants were calculated according to the following mathematical formula:

(1) $$k=\frac{2.3}{Bt}log\frac{P0}{P}$$

where k is the adsorption rate constant (in mL/min); *B* is the concentration of bacterial cells; and *t* is the time interval in which the titer falls from *P*0 (original phage titer) to *P* (final phage titer) [41].

### 2.7. Long-Term Study of Phage Stability under Various Temperature Conditions

Phage lysates in the determined titers (1.62 × 10^10^ PFU/mL for Entb_43; 3.67 × 10^7^ PFU/mL for Entb_45—the difference is that a higher titer could not be obtained at the beginning of the experiment) were placed in different temperature conditions: 37 °C, 22–24 °C (room temperature—RT), 4 °C, and frozen at −70 °C with or without addition of 20% glycerol (Difco). Experiments were performed over 6 months, and samples were taken after 1, 2, 4, 10, 14, 18, and 24 weeks, respectively. Additionally, to assess if defrosting affected the titer of bacteriophages stored at −70 °C, the same sample was defrosted several times at different time intervals. Phage titers were determined using RTD and the double-layer agar method in triplicate.

### 2.8. Investigation of Phage Stability in Solutions of Different pH and Disinfectants

To evaluate the stability of phages in solutions with a different pH, we tested the liquid culture medium—peptone water at a different pH, at a range from 3 to 12 (established with 5 M HCl or 10 M NaOH). Phage suspensions in pH = 7.2 (standard culture medium) were used as controls. For this purpose, 200 µL of phage lysate (10^9^ PFU/mL) was added to 1800 µL of a specific solution (at 1:9 ratio) and incubated for 1 h at 37 °C in a water bath. After this period, serial dilutions of phages were prepared, and their titers were determined using RTD in triplicate. In addition, the stability of bacteriophages in various disinfectants or surfactants (including 70% ethanol, 10% dish soap, 10% liquid soap, 10% hand wash gel, and two antibacterial and antiviral liquid skin disinfectants diluted 2 times) was assessed. Using a similar method to that presented above, 200 µL of phage lysates were added to 1800 µL of specific solution and incubated at room temperature. Phage titers were determined after 5, 10, 20, and 30 min of incubation. All experiments were performed in triplicate. Results are presented as a percentage of active phages [40]. A similar method was used to test the phage stability with commercially available colloidal silver and copper solutions (Nano-Tech, Polska) at a concentration of 50 mg/kg (ppm), also used as antibacterial and antifungal agents. In this case, an additional 24 h of incubation was performed.

### 2.9. Stability of Phage Lysates in Urine Sample In Vitro

The fresh urine sample collected from the midstream was obtained from a healthy donor. The urine sample presented pH = 6, and a density of 1.015. The glucose, ketones, proteins, and nitrites were absent from the urine sample, and flat epithelial cells were sparse. The sample was filtered through 0.22 µm pore size filters. As in previous experiments, phages were incubated with the sample with a ratio of 1:9 at 37 °C for 30 min, 1 h, and 24 h. Phage titers after defined incubation times were determined by RTD. The experiment was performed in triplicate. Phage titer at the start of the experiment (time zero) was used as a comparison for the statistical analysis.

### 2.10. Transmission Electron Microscopy (TEM)

Phage lysates were prepared in a LB (Luria–Bertani) medium in high titers (Entb_43: 2.3 × 10^10^ PFU/mL; Entb_45: 1.22 × 10^9^ PFU/mL) and filtered two times. Samples were concentrated using ultracentrifugation (1 h, 7 °C, 25,000 × *g*) and, after removing the supernatant, 0.1 M ammonium acetate was added and centrifuged in the same conditions. In the next step, a drop of phage lysate was placed on a 400 mesh copper grid (Athene). After being contrasted with 2% uranyl acetate, preparations were dried and analyzed using the Zeiss EM900 TEM (at an acceleration voltage 80 kV). Images of phages were obtained using Kodak/Carestream Electron Microscope Film 4489. Phages’ morphology and dimensions were defined.

### 2.11. Phage DNA Isolation Procedure and DNA Sequencing

Phage lysates at a volume of 20 mL in high titers (Entb_43: 2.3 × 10^10^ PFU/mL; Entb_45: 1.22 × 10^9^ PFU/mL) were concentrated with the use of Amicon Ultra Centrifugal Filters (Merck, NJ, USA). To isolate the phage nucleic acid (dsDNA), QIAamp MinElute Virus kits (Qiagen, Germantown, MD, USA) were used. Analysis of dsDNA concentrations was prepared using QuantiFluor dsDNA System (Quantus, Promega, Madison, WI, USA), which uses a fluorescent DNA-binding dye. The Illumina DNA Prep system was used to construct the sequencing library, following the assessment of their quality using a high sensitivity D1000 ScreenTape Assay for TapeStation System (Agilent, Santa Clara, CA, USA). Sequencing of phage genomic DNA was performed with MiSEQ Reagent Kit Nano V2 or MiSEQ Reagent Kit V3 (New Generation Sequencing, Illumina system).

Read sequences (fastq) (from Illumina technology sequencing) for both phages were subjected to the same general pipeline. Quality assessment was completed with the Trimmomatic software [42]. Reads passing the quality check were used by SPAdes software (3.12) to assemble reads into contigs and scaffolds [43,44,45,46,47]. For the Entb_43 phage, k-mer size was detected automatically, but for Entb_45 it was set to 21, 33, and 55 with additional coverage cutoff set to 50. Assembly artifacts were checked and removed if recognized as bacterial contamination. This allowed one dominant scaffold to be assembled which relates to other phages, and this was used as a partial genome for further analysis. BLASTn (megablast) was used to evaluate similarity to prokaryotic and phage genomes in currently existing databases.

Genome annotations were performed with the Prokka software [48,49] and PGAP (Prokaryotic Genome Assembly Pipeline) [50,51,52] using prokaryotic databases. This forced adherence to International Nucleotide Sequence Database Collaboration (NCBI, ENA and DDBJ) standards and up-to-date databases. The PGAP annotation process is described in detail on the NCBI webpage (https://www.ncbi.nlm.nih.gov/genome/annotation_prok/process/, accessed on 12 May 2022) and both processes result in functional annotation of genomes. In short, this approach mixes alignment-based methods, functional assessments of elements directly from the sequence (with the help of hidden Markov Models), representative reference proteins/genomes, and software (such as GeneMarkS2+). Alignments of closely related genomes were calculated by Geneious Prime 2022.1.1 (http://www.geneious.com, accessed on 12 May 2022) [53] and progressiveMauve [54]. SD motifs (ribosome binding sites) were found by the XSTREME [55] tool connecting the MEME, STREME, and SEA software to perform comprehensive motif discovery in accordance with guidelines for bacteriophage annotation [56].

### 2.12. Statistical Analysis

The statistical analysis was performed using the GraphPad Prism 9 software. A one-way ANOVA was used to determine if any changing factors (stability in nanoparticles, in solutions with different pH and in urine) influenced the phage titer and a Dunnett’s multiple comparisons test determined the statistical significance of differences between groups. Statistical significance was considered at *p* < 0.05. All experiments were performed in at least three repetitions.

## 3. Results

### 3.1. Characterization of the Bacterial Strains

The twenty bacterial strains used in the experiments were mainly identified as *E. cloacae* (15/20), but also as *E. hormaechei* (4/20) and *E. kobei* (1/20) (Table 1). All tested strains were resistant to amoxicillin/clavulanic acid. The host strains turned out to be multidrug resistant (resistance to several antibiotics simultaneously)—e.g., *E. cloacae* 30345, which has been shown to be resistant to amoxicillin/clavulanic acid, ciprofloxacin, levofloxacin, and tobramycin, and *E. cloacae* 29796, which, in addition to the antibiotics mentioned above, is resistant to cefotaxime, cefepime, gentamicin, and trimethoprim/sulfamethoxazole. Single bacterial strains also showed resistance to fosfomycin (*E. cloacae* 3344 and *E. hormaechei* 30550), as well as to a wider range of antibiotics from different pharmacological groups—e.g., ciprofloxacin, cefepime, ertapenem, gentamicin, levofloxacin, piperacillin/tazobactam, and tobramycin (*E. cloacae* 30342).

### 3.2. Isolated Bacteriophages

After screening over 300 environmental samples, we isolated three new, potentially lytic bacteriophages, specific for *Enterobacter* spp.: Entb_43 (vB_EclM-43), Entb_44 (vB_EclM-44), and Entb_45 (vB_EclM-45). As a result of genetic analysis, we proved that the Entb_44 phage has an almost identical genome to Entb_43, so we decided to further characterize only the Entb_43 and Entb_45 phages. Both phages show species-specificity for *Enterobacter* spp. only—no lysis of other Gram-negative and Gram-positive strains from the ESKAPE group was observed (Table 2). The broadest lytic spectrum was determined for the Entb_45 phage, which also had the highest species specificity—causing lysis on various species belonging to *Enterobacter* (*E. cloacae*, *E. hormaechei*, *E. kobei*) (Table 3).

For two strains, which were hosts for phages: *E. cloacae* 30345 (host for Entb_43) and *E. cloacae* 29796 (host for Entb_45), plots of dependence of the bacterial titer (CFU/mL) on the OD_600_ were prepared.

### 3.3. Morphology of Entb_43 and Entb_45 Phages and Their Plaques

Bacteriophage Entb_43 formed small, clear plaques on the solidified agar plates (less than 1 mm in diameter) with no apparent “halo” effect. The Entb_45 phage created slightly larger clear plaques on the agar plates (approximately 1–2 mm in diameter), and after about 3 days a slight “halo” effect was noticeable.

**Figure 1:**
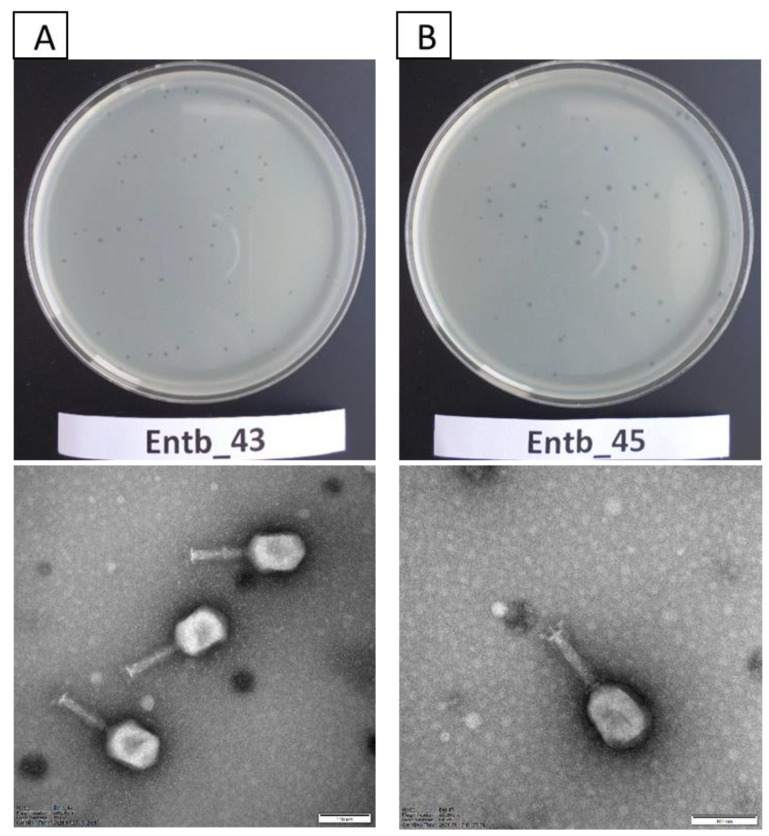
Plaque morphology on agar plates (upper panel) and electron micrographs of phages (lower panel): (**A**) phage Entb_43 and (**B**) phage Entb_45. The photographs of the phages’ plaques were taken using a Samsung ST150F camera. Electronograms of the phages were taken using Kodak/Carestream Electron Microscope, at a magnification of (**A**) 200,000× or (**B**) 250,000×; bar in each micrograph indicates 100 nm.

Studies with the use of TEM revealed that both newly isolated bacteriophages had the A2 morphotype. The morphology of the phages corresponds to that of myoviruses (Figure 1), which are characterized by contractile tails and icosahedral heads [57,58,59]. Entb_43, but not Entb_45, had a visible belt structure at the level of one-third of the tail. Dimensions of the capsids, tails, and base plates of each phage are included in Table 4.

### 3.4. Influence of Different MOI on the Effectiveness of Phage Amplification and Antimicrobial Effect

As shown in Table 5, the phage titers, after overnight cultivation at 37 °C, varied depending on the phage-to-bacteria ratio used (different MOI). In general, the lower MOI used (lower phage titer than bacterial titer), the higher final phage titer obtained. For both phages, the best phage-to-bacteria ratio was either 0.01 or 0.001.

**Figure 2:**
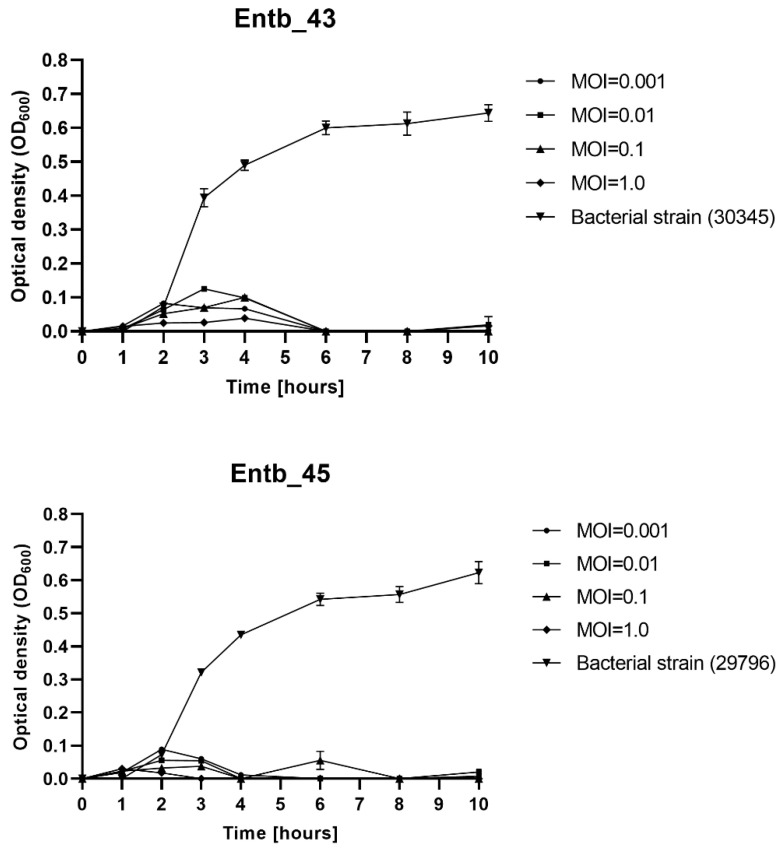
Planktonic cell lysis assay for phage Entb_43 and phage Entb_45. Sample measurements were performed in triplicate in two independent experiments. Error bars represent the standard deviation (±SD) of the mean phage titers.

The MOI-dependent ability of phages to lyse planktonic bacteria was determined. As shown on Figure 2, each of the bacteriophages significantly inhibited the growth of the host bacteria. Bacterial lysis varied slightly on the used MOI; however, even a low MOI is sufficient to successfully eliminate bacterial cells, whereas the OD_600_ of the phage-untreated bacterial strains (positive controls) clearly increased over time. The optical density of the only phage lysates (negative controls) oscillated around 0.00 and did not change during the measurements (not shown in the graphs).

### 3.5. Phage Adsorption to Bacterial Cells

**Figure 3:**
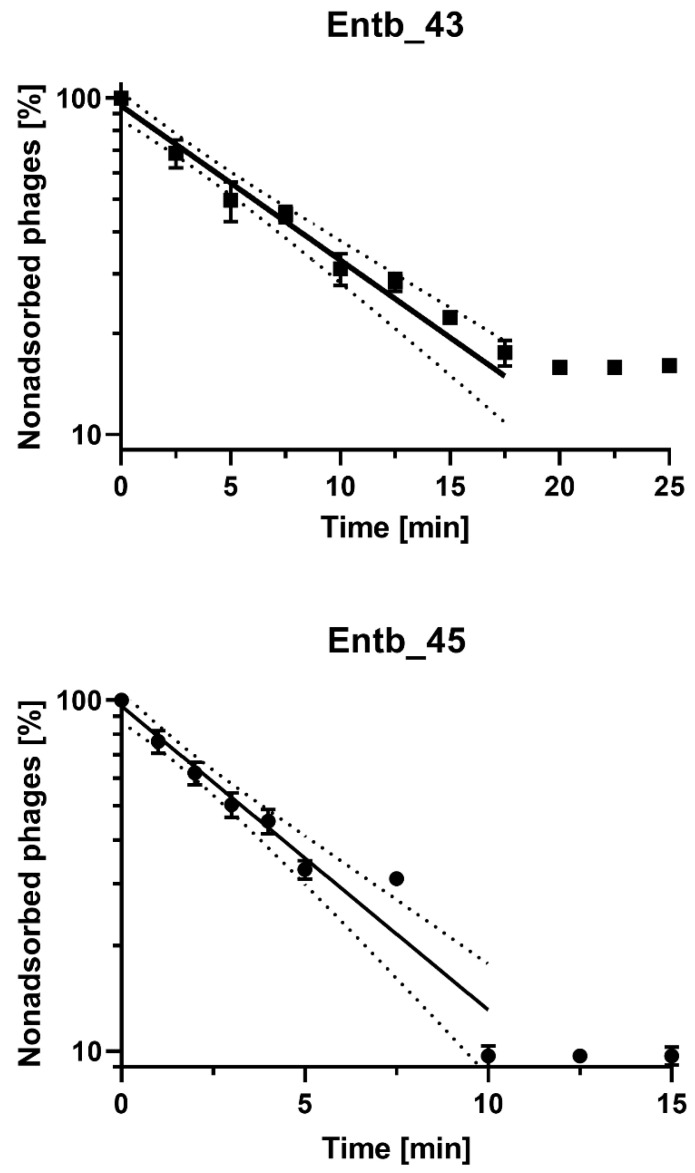
Kinetics of Entb_43 phage adsorption on *E. cloacae* 30345 and Entb_45 phage on *E. cloacae* 29796 at MOI = 0.1. Error bars represent the standard deviation (± SD) of the mean phage titers. Lines represent model used for calculation of adsorption constant; dotted line is 95% confidence interval (CI).

After the infection of *E. cloacae* 30345 with the Entb_43 phage (at MOI = 0.1), we obtained about 84% adsorption of the phage particles to the host cells at 37 °C in 20 min. However, the adsorption time of Entb_45 to *E. cloacae* 29796 was much faster, and after 10 min, almost 90% adsorption was achieved (Figure 3 and Supplementary Figure S1). The adsorption rate constants, which are included in Table 6, were also calculated.

### 3.6. Genome Sequencing

We present the genomes of two new phages: *Enterobacter* phage Entb_43 (174 kbp, acc. No. ON585039) and *Enterobacter* phage Entb_45 (173 kbp, acc. No. ON630910). Both phages were sequenced with Illumina technology and assembled into a single, gapless contig from reads passing through a quality check (see Materials and Methods for details). Phage genomes show a high degree of diversity between each other, with only 12% of the genome showing high similarity between two of them. These similarities were, however, limited (up to 75% of identity) and they were found mainly in regions coding for structural phage proteins. The presented genomes show poor genomic similarity to already known phages. In databases accessible through the International Nucleotide Sequence Database Collaboration (https://www.insdc.org/, accessed on 10 June 2022), only 8 and 7 phage genomes show a similarity above 70% to Entb_43 and Entb_45, respectively (Supplementary Tables S1 and S2). The first one is most closely related to the *Klebsiella* phage vB_KaeM KaAlpha (93% identical at the nucleotide level, acc. no. MN013084) and the second one is most closely related to the *Enterobacter* phages ENC9 (93% identical at the nucleotide level, acc. no. OL355124.1) and vB_EclM_CIP9 (94% identical at the nucleotide level, acc. no. NC_048849.1). Basic features of the presented phages are described in Table 7.

For the genomes of Entb_43 and Entb_45, revealed after annotation 298 and 289 coding sequences, respectively, 44% and 58% of the proteins are designated as “hypothetical proteins”. One hypothetical protein is present in a tail gene cluster of Entb_43, but not in the Entb_45 phage (protein ID: M8345145). The highest similarity (except for hypothetical proteins) is shown for a putative GIY-YIG homing endonuclease (*Yersinia* phage vB_Yem_TG1, acc. no.: NC_028820.1), but no significant similarity to well-described proteins was found. Typically for *Tevenvirinae*, our annotation in both phages revealed no apparent integrase gene, suggesting a lytic life cycle. Genomes were linearized to the presumed position at the beginning of the identified gene coding the rIIA/B gene, as is standard for *Tevenvirinae*. Analysis of the Entb_43 genome revealed 19 tRNA, but only 9 tRNA in Entb_45. No similarity to antibiotic resistance genes were found in the CARD database (no strict hits) [60].

**Figure 4:**
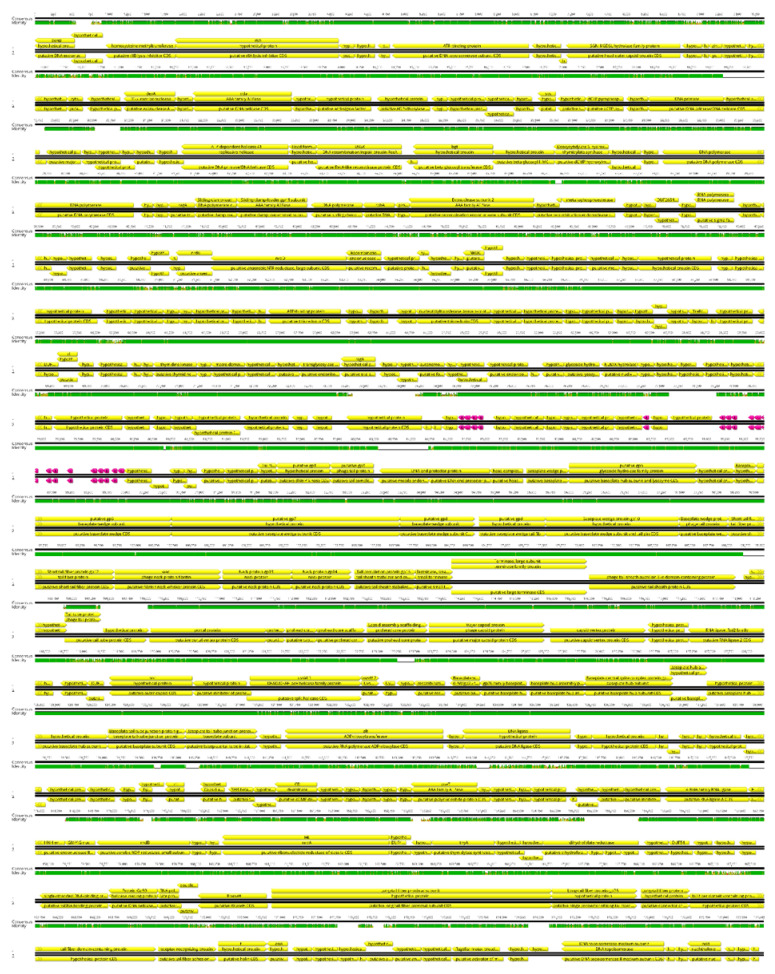
Graphical representation of phage Entb_43 (no. 1), aligned to the most similar known genome of *Klebsiella* phage vB_KaeM KaAlpha (no. 2, acc. no. MN013084). Green—100%, brown-green—between 30% and 100%, and red—0% local similarity. Lack of color shows a gap in alignment. Annotation is shown for reference. Coding sequences (CDS) are yellow, and tRNA coding sequences are pink.

**Figure 5:**
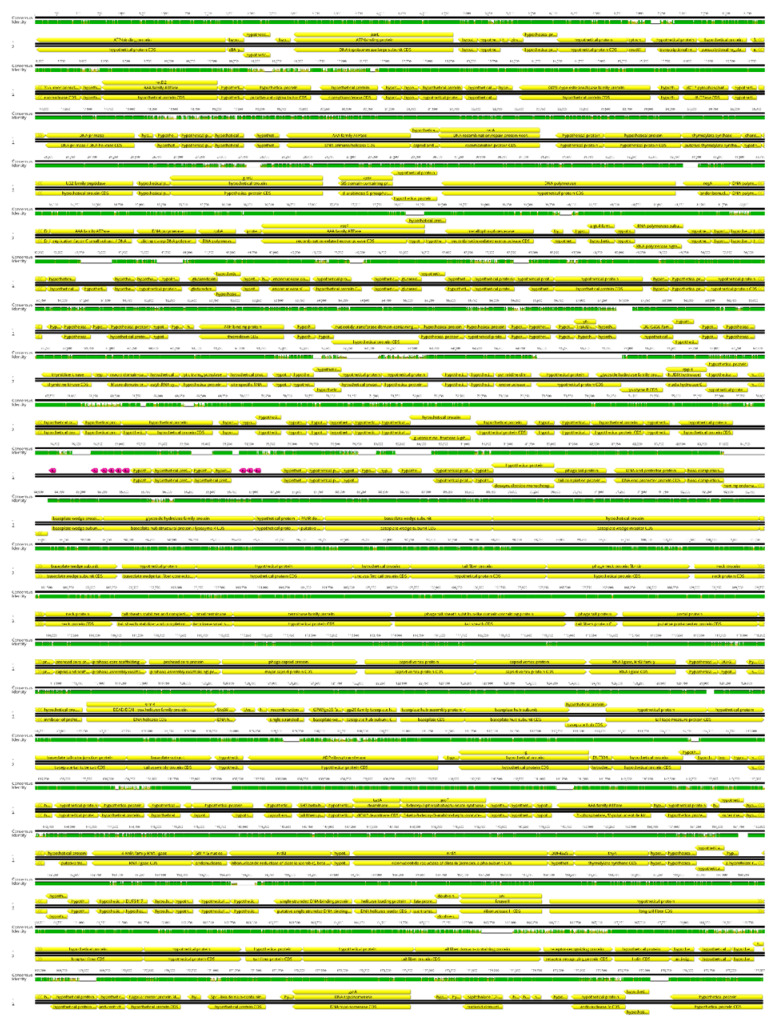
Graphical representation of phage Entb_45 (no. 1), aligned to the most similar known genome of *Enterobacter* phage ENC9 (no. 2, acc. no. OL355124.1). Green—100%, brown-green—between 30% and 100%, and red—0% local similarity. Lack of color shows a gap in alignment. Annotation is shown for reference. Coding sequences (CDS) are yellow, and tRNA coding sequences are pink.

**Figure 6:**
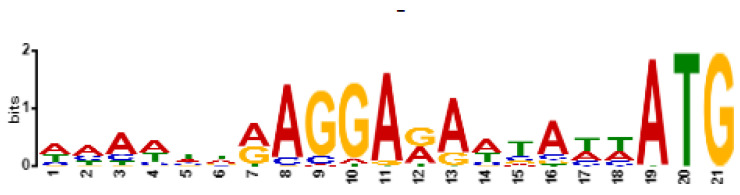
Shine–Dalgarno motif (ribosome binding site) in sequences of 100 bp upstream of START codon (included) for phage Entb_43, identified by XSTREME software.

**Figure 7:**
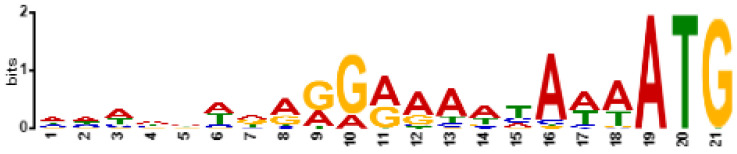
Shine–Dalgarno motif (ribosome binding site) in sequences of 100 bp upstream of the START codon (included) for phage Entb_45, identified by XSTREME software.

Based on genomic similarity, we classified phage Entb_43 to genus *Karamvirus* and Entb_45 phage to genus *Kanagawavirus* [61,62], which classifies both phages to the *Tevenvirinae* subfamily (*Straboviridae* family) in accordance with current ICTV guidelines [63]. Each phage genome showed a similarity of over 70% to representatives of only one genus. Similarity to the most closely similar phage is shown in Figure 4 and Figure 5. Similarity of the marker genes recommended by ICTV for *Tevenvirinae* shows that Entb_43 and Entb_45 are closely related to other representative phages from the *Karamvirus* and *Kanagawavirus* genera, respectively (Supplementary Tables S3 and S4) [64]. We identified Shine–Dalgarno sequences (ribosome binding site) in fragments 100 bp upstream of the START codon (Figure 6 and Figure 7) according to the recommendation by Turner et al. in the “Phage Annotation Guide: Guidelines for Assembly and High-Quality Annotation” [56].

### 3.7. Stability Studies of New Enterobacter-Specific Phages

In order to assess the effect of commonly used disinfectants and cleaning agents on the stability of bacteriophages, six different commercially available substances were used in the experiment (Table 8). In general, a significant reduction in phage titers was observed after incubation with the disinfectant that contained the active substances octenidinum dihydrochloridum and phenoxyethanol (1) (titer reduction of over 90% for phage Entb_43 and by 99.9% in the case of phage Entb_45, after 30 min of incubation), and was slightly less pronounced with 70% ethanol (reduction in the titer by nearly 70% for Entb_43 and by 97% for phage Entb_45, after 30 min of incubation). The remaining disinfectants and surfactants influenced the phage titers, but this was less significant.

**Figure 8:**
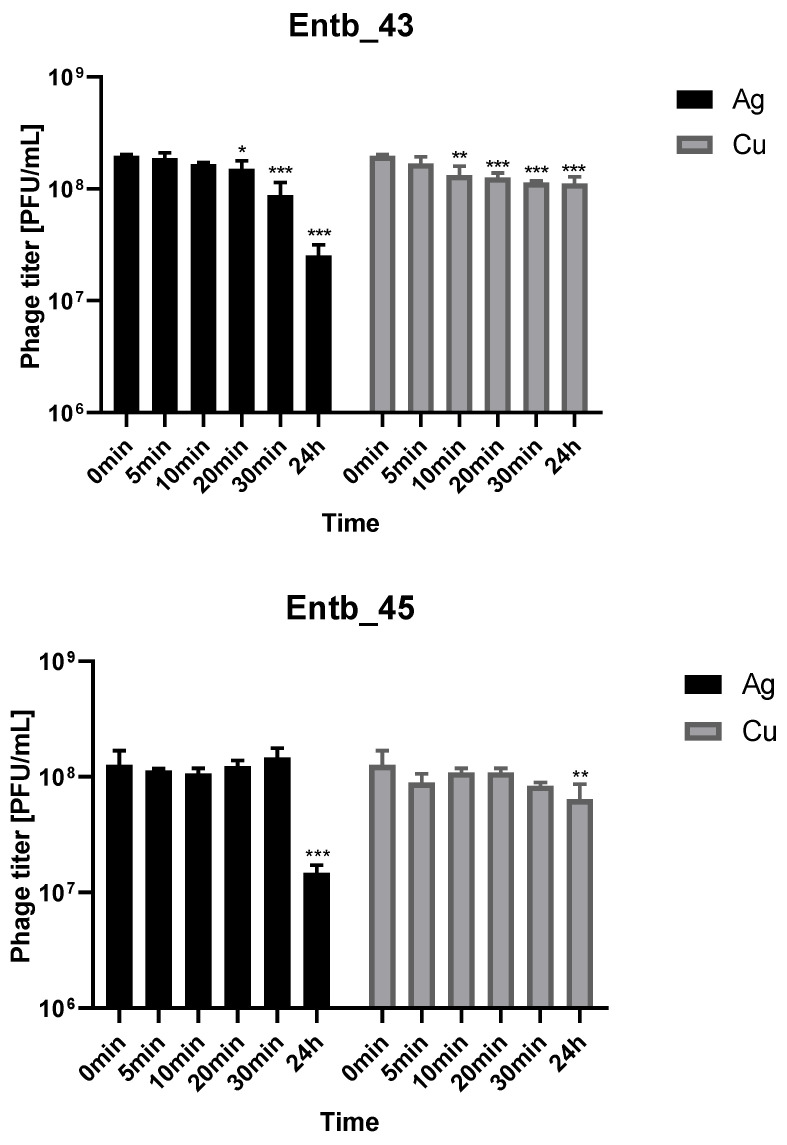
Phage lysate stability in silver nanoparticles and copper nanoparticles. Samples at the start of the experiments (0 min) were treated as controls for statistical analysis. Error bars represent the standard deviation (±SD) of the mean phage titers. * *p* < 0.05; ** *p* < 0.01; *** *p* < 0.001.

Metal nanoparticles such as copper and silver are used in a variety of ways as disinfectants. We decided to evaluate the stability of phage lysates in these solutions to assess if applying them together was possible. As shown in Figure 8, both bacteriophages retain a lot of active particles for 30 min of incubation with nanoparticle solutions at room temperature. On the other hand, 24 h incubation caused more significant reductions in phage titers in the case of silver colloid than copper.

**Figure 9:**
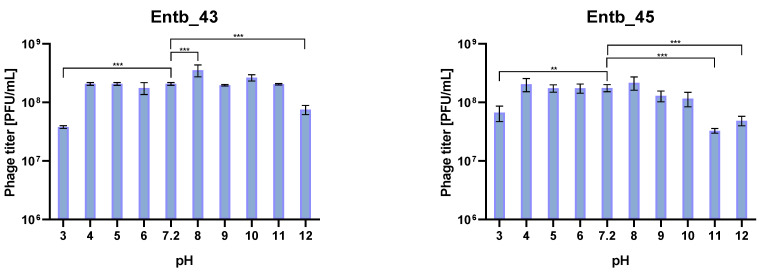
Phage titers after one-hour incubation in different pH solutions. Samples incubated in a medium with pH = 7.2 were treated as controls. Error bars represent the standard deviation (±SD) of the mean phage titers. ** *p* < 0.01; *** *p* < 0.001.

The isolated bacteriophages were also tested for stability in the culture medium (peptone water) with different pH values. As a control for comparisons, samples with peptone water with pH = 7.2 were used, which were treated the same as the other samples. As a result of the statistical analysis, it was observed that the most significant differences in phage titers after incubation occurred in the case of the most extreme pH values (pH = 12 for phages Entb_43 and Entb_45, pH = 11 for phage Entb_45, and pH = 3 for both phages). Interestingly, higher phage titers were obtained as a result of incubation with a medium for which the pH was 8, which may suggest more effective results of phage amplification in slightly more alkaline media than before (Figure 9).

**Figure 10:**
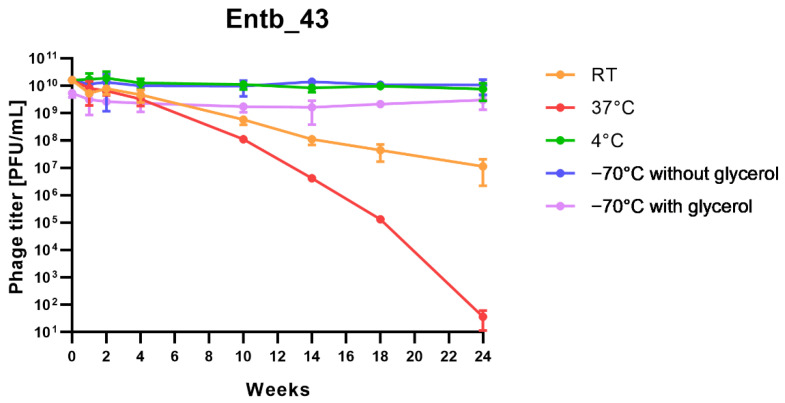

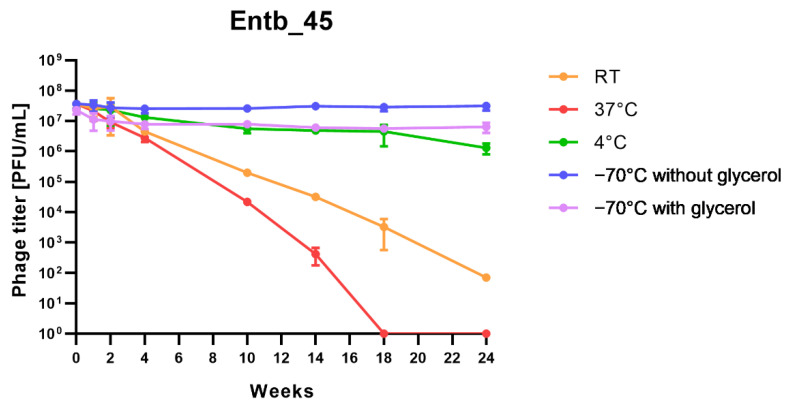
Long-term stability of Entb_43 and Entb_45 phage lysates under various temperature conditions. Error bars represent the standard deviation (±SD) of the mean phage titers.

**Figure 11:**
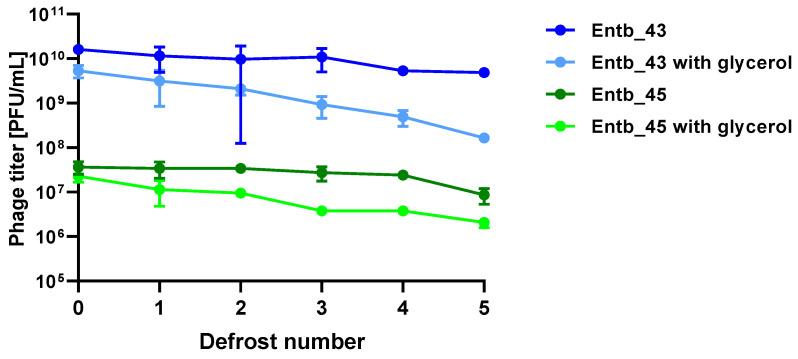
Influence of repeated defrosting on titers of phage lysates with or without 20% glycerol. Error bars represent the standard deviation (±SD) of the mean phage titers.

Stability of phages over six months (24 weeks) was clearly dependent on the temperature at which they were stored. As was shown in Figure 10, storage of bacteriophages at 4 °C, as well as in frozen form at −70 °C (with or without the addition of cryoprotective glycerol), did not significantly affect their titers (only the titer of Entb_45 stored at 4 °C, which initially had a lower titer, decreased by one order of magnitude on a logarithmic scale at the end of the experiment). Phage titers decreased successively at both room temperature and 37 °C. No active phage particles were found in the Entb_45 phage lysate after 18 weeks of storage at 37 °C, whereas the titer of the Entb_43 phage decreased from 10^10^ to 10^1^ PFU/mL after 24 weeks. Moreover, the thaw-resistance of frozen phage lysates was demonstrated, because after defrosting the same samples five times and refreezing, the phage titers only decreased by about one order of magnitude (Figure 11).

### 3.8. Phage Stability in a Urine Sample

**Figure 12:**
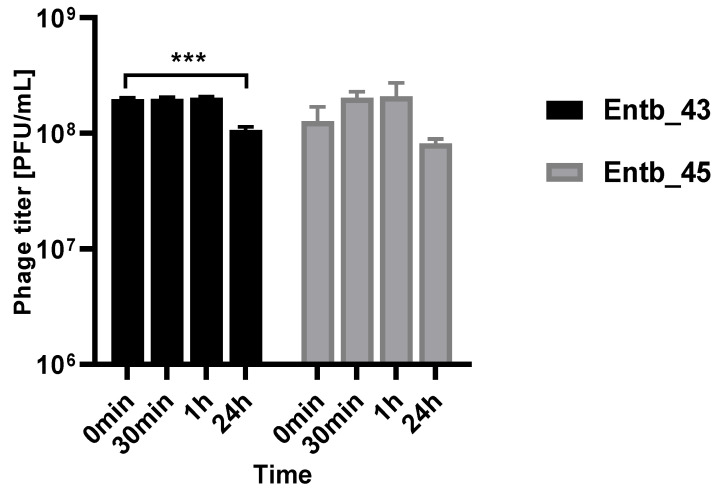
Phage titers after incubation in a urine sample (after 30 min, 1 h, and 24 h of incubation at 37 °C). Samples at the start of the experiments (0 min) were treated as controls. Error bars represent the standard deviation (± SD) of the mean phage titers. *** *p* < 0.001.

As mentioned before, *Enterobacter* spp. can cause severe urinary tract infections. A key feature of phage therapy in the intravesical administration of phages is their stability at the site of action, so we examined their ability to remain active in a urine sample (pH = 6) obtained from a healthy donor (Figure 12). It was noted that, after both half an hour and an hour of incubation in a fresh sample of urine, the phage titers remained at the same level as at the beginning of the experiment, which may suggest their potential efficacy without loss of activity at the infection site after intravesical administration.

## 4. Discussion

Because *Enterobacter* strains are one of the etiological factors of various life-threatening conditions, we decided to isolate and characterize new phages specific to these bacteria. Our characteristics concerned not only the standard life cycle parameters, the lytic spectrum, and the stability under various conditions, but also the detailed genomic characteristics (as suggested by Suh et al., 2021 [65]).

As a result of typing 339 samples from various sources, we were able to find three new bacteriophages (Entb_43, Entb_44, and Entb_45) showing an ability to lyse clinical *Enterobacter* strains. Despite the fact that the bacteriophages Entb_43 and Entb_44 were isolated from different environmental samples, a genetic analysis showed that they were too similar to be considered separate phages. Therefore, we decided to further characterize only Entb_43 and Entb_45.

Currently, 85 genomes of *Enterobacter* phages have been deposited in the gene bank (https://www.ncbi.nlm.nih.gov/nuccore, accessed on 26 May 2022), which may indicate the potential and the need to conduct research on these phages and their comprehensive characteristics. Bioinformatic analysis of the genomes of two of our new bacteriophages allowed us to describe them as double-stranded DNA and to determine their sizes, respectively, as 174,681 bp for Entb_43 and 172,771 bp for Entb_45. Similar results have already been reported for the *Enterobacter*-specific phage vB_EclM_CIP9 (defined as myovirus) with a 174,924 bp genome [66], as well as the EBP bacteriophage (also myovirus), which has a 179.1 kb genome size [67]. Interestingly, a phage active against *E. cloacae* was recently reported with a genome size of 51,894 bp and was assigned to a new genus, *Eclunavirus* [68]. Moreover, no rRNA or tRNA were found in its genome, whereas our phages had 19 and 9 tRNAs in their genomes, respectively. The similarity of Entb_43 to the *Klebsiella*-specific phage may be related to the similarity of these two types of bacteria. Until recently, *Klebsiella aerogenes* was considered *Enterobacter aerogenes* [9,69]. Moreover, genomic analyses provide data on the genetic relationships of these bacteria, as described, for example, for strains isolated from patients with a bloodstream infection (69% shared genes) [70].

The host range of phages estimated on the basis of the spot test was 40% and 60%, respectively. Both phages were also tested simultaneously: immediately after mixing, 24 h after, 48 h after, and one week after mixing, but no significant changes in the lytic spectrum were observed. The phage cocktail retained its lytic properties on strains against which at least one phage was active (data not presented herein). Interestingly, the myPSH1140 phage, described by Manohar et al. (2019), showed a much wider host range, reaching 100% of *E. cloacae* and 75% of *E. hormaechei* strains [71]. There are also *Enterobacter*-specific phages with an extremely narrow range of activity, lysing only one bacterial strain [72]. Instead, phages are also described as polyvalent, as presented by Finney et al. (2022), with phages lysing both *Enterobacter* spp. and *Klebsiella aerogenes* [73], or by Addablah et al. (2021), with phages infecting *E. cloacae* and *E. coli* simultaneously [74]. Jamal et al. (2018) reported the phage MJ2, which was able to inhibit the growth of *E. cloacae*, *Achromobacter xylosoxidans*, *K. pneumoniae*, and *P. aeruginosa* [75]. However, determination of the host range is not always clear and repeatable due to the different determination methods and the ability of phages to form plaques on the specific bacterial lawn [76].

Similar morphological characterization, as well as host range, was described for a lytic EBP bacteriophage by Asif et al. (2021) [67] and for three phages reported by Gibson et al. (2019) [77]. Nair et al. (2021) also described a myovirus, which was able to lyse both the planktonic form of bacteria and the biofilm produced by *E. cloacae* [78].

In the adsorption experiment, we used MOI = 0.1 for both phages (10^7^ PFU/mL and 10^8^ CFU/mL) and observed that phages achieved complete adsorption to host cells in 20 min (Entb_43) and 10 min (Entb_45). Conversely, phages that show more rapid adsorption were also described, such as the *E. coli*-specific phages vB-EcoS-95 (50% adsorbed phage particles in 2 min) [79] and vB_Eco4M-7 (completed adsorption of 90% after 1 min) [40], or the *Klebsiella*-specific phages (4 min and 6 min) [39]. Statistical analysis allowed us to define adsorption as a first-order kinetic process [41] (Figure 3), even though basic graphs on a nonlogarithmic scale might indicate biphasic adsorption (Supplementary Figure S1).

The best phage-to-bacteria ratio, both for estimating the optimal conditions for bacteriophage amplification and for lytic activity against planktonic bacteria, turned out to be 1:1000 or 1:100 (MOI = 0.001 or MOI = 0.01). Interestingly, studies using the new phage against *Enterococcus faecalis* producing biofilm in Foley catheters initially reported more efficacy of phage lytic activity at a higher MOI (MOI = 10). On the other hand, after 6 h and 24 h of exposing the bacterial biofilm to the phage, the preparations with a lower phage-to-bacteria ratio (MOI = 0.01 and MOI = 0.0001) were more effective [80]. However, we describe the MOI as the ratio of the number of phages to the number of bacteria at the start of the experiment, and it is suggested to also consider, inter alia, the changing densities of bacteria during the course of the experiment [81].

In the case of long-term storage of phage preparations, a temperature of 4 °C is usually recommended [82]. Our phage lysates (in peptone water) remained stable at this temperature throughout the six-month period of the experiment. The maintenance of stability by phages in frozen forms (−70 °C) indicates such a possibility for their storage. Interestingly, over the course of a month, only the titers of phages stored at the highest temperature (37 °C) decreased by only one order of magnitude. It is worth noting that studies on adaptive evolution regarding various thermal conditions are being designed to increase phage stability [83].

The ability of phages to remain active under unfavorable conditions may be very important for people working with these viruses, as well as for the industrial or pharmaceutical applications of phages [84]. An example of the industrial use of *E. cloacae*-specific bacteriophages is the attempt to use them as factors to reduce the bloating defect caused by these bacteria in cucumber fermentation [85]. The different values of acidity/alkalinity of the environment are of particular importance in the context of the human organism and other animals in the processes of health and disease. From the point of view of phage therapy, factors such as changes in the pH of skin and wounds [86], unfavorable pH in the stomach [87], or changes in the pH of the environment caused by growth of bacteria [88] can determine the effectiveness of treatments. Relatively good stability was observed in our phages after incubation in peptone water with a pH ranging from 4 to 11. Moreover, the observed decrease in titers as a result of a large deviation from physiological pH has been confirmed for most phages, including *Enterobacter*-specific [71,89,90,91,92] and other species (e.g., *Escherichia coli*-specific) [93]. Very similar tolerance to a wide pH range was also shown by the phages KKP 3262 and KKP 3263 described by Wójcicki et al. (2021) [94]. Interestingly, two of five *E. hormaechei*-specific phages isolated by Chen and coworkers (2022) showed an almost complete resistance to a more alkaline environment (such as pH = 12) [95].

The inactivation of phages as a result of contact with various disinfectants/surfactants may be of great importance in the quality of work involving them, and may also have an impact on the field of therapeutics when the use of surface disinfectants can support phage action (e.g., skin surface disinfection or coating the surface of catheters with metal nanoparticles). Compounds based on silver were applied in antimicrobial therapy until the 1940s when antibiotics were implemented [96]. The observed antimicrobial activity of silver may be the result of a direct toxic effect on bacterial cell walls [97]. When silver ions are released, the bacterial cell membrane and a variety of intracellular functions are disrupted. The small size of their particles, together with the large surface area of the dressing’s integrated silver nanoparticles, increase their solubility and cause a sustained release of silver ions in relatively nontoxic concentrations [96]. Coating the catheter’s surface with silver nanoparticles resulted in significant in vitro antimicrobial activity and also prevented biofilm formation by *E. coli*, *Enterococcus*, *S. aureus*, coagulase-negative staphylococci, *P. aeruginosa*, and *Candida albicans* [98]. Studies by Thomas et al. (2015) indicate a high potential in applying silver nanoparticles to urinary catheters for preventing catheter-associated infections, especially the attachment and colonization of bacteria [99,100]. The scientific literature also indicates that copper nanoparticles have antibacterial properties [101,102]. Therefore, it is important to evaluate phage–nanoparticle interactions due to data about the possibility of irreversible inactivation of phages (e.g., *E. coli*-specific) by nanoparticles [103,104]. The activity of our new phages, despite a statistically significant difference compared to the beginning of incubation, was well maintained for about 30 min of incubation at RT with both types of nanoparticles. This may indicate the possibility of a synergistic antibacterial effect and the need for further research into this phenomenon. Similarly, Abdelsattar et al. (2021) showed no significant differences in the titer of a *Salmonella*-specific phage after 4 h incubation at 37 °C with synthesized silver nanoparticles [105]. The 24 h incubation, especially in the case of silver, led to a more significant inactivation of our phages.

We also decided to test the phage stability in 10% dish soap and 10% liquid hand soap. In similar experiments, Necel et al. (2020) [40] found no decrease in two *E. coli* phage titers after 2 min and 5 min of incubation at RT, respectively. Our results indicated an initial reduction in titers for both phages by approximately 20–25% (after 5 min), while for half an hour, despite fluctuations in the titers, no reduction was observed. The commonly used skin disinfectant based on octenidinum dihydrochloridum and phenoxyethanol, as well as 70% ethanol, had the strongest effect in reducing the number of active phage particles, which may be important in skin or wound disinfection before phage administration. For ECML-117 and vB_Eco4M-7 phages, active phage particles were described as 0% and 0.01%, respectively, after one hour of incubation in 63% ethanol [40]. Likewise, such conditions caused a significant decrease in vB-EcoS-95 phage survival (0.8% active particles) [79]. Interestingly, Tomat et al. (2018) [106] reported *E. coli* phages that remained active after 24 h of incubation in 100% ethanol (decrease of titer by 2.5 PFU/mL). Recently, the possible negative effects of the uncontrolled spread of phages in food production processes have also been emphasized [107]. Information about the factors that deactivate them may therefore be useful in industrial fields.

Beta-lactam antibiotics, as well as fluoroquinolones and aminoglycosides, are the most common antimicrobials prescribed to treat infections caused by *Enterobacteriaceae* [108]. The bacteria we used demonstrated ESBL+ resistance, a mechanism that is characteristic of uropathogenic *Enterobacter* strains [109,110] and other Gram-negative bacteria [111]. More relevant data are emerging on the prospects of managing UTIs through the use of lytic bacteriophages, phage cocktails, enzymes derived from them, and genetically modified phages, as well as by using the synergistic effect of phages and antibiotics [112,113,114,115,116]. Promising clinical trials in this area are slowly emerging [117,118], but many issues still need to be refined [119]. There are reports of clinical successes in the treatment of UTIs as a result of intravenous phage cocktail administration, which, although it did not completely eradicate the pathogen, its succession resulted in a significant improvement in the patient’s condition [120]. As a preliminary study determining the possibility of using our phages in the treatment of UTI, we decided to evaluate their stability in a urine sample. The results turned out to be promising as bacteriophages were not inactivated by the components of the previously filtered urine. Pereira et al. (2016) described not only the good stability of *Enterobacter*-specific phages in a urine sample, but also an increase in their titer in the presence of bacteria [113].

The results described in this paper strongly suggest the therapeutic potential of the presented *Enterobacter*-specific bacteriophages.

## Figures and Tables

**Table 1:** Bacterial strains used in the experiments.

Strain Number	Name of Bacterial Strain	Source of Isolation
1.	*Enterobacter cloacae* 30345	fistula
2.	*Enterobacter cloacae (ESBL+)* 29796	urine
3.	*Enterobacter cloacae* 3344	urethra
4.	*Enterobacter cloacae* 3345	foreskin
5.	*Enterobacter cloacae* 30697	semen
6.	*Enterobacter cloacae* 30528	pus
7.	*Enterobacter cloacae* 30103	pus
8.	*Enterobacter cloacae* 29916	pus
9.	*Enterobacter cloacae (ESBL+)* 29779	pus
10.	*Enterobacter cloacae (ESBL+)* 29731	sore
11.	*Enterobacter cloacae* 30642	foot ulcer
12.	*Enterobacter cloacae (ESBL+)* 30256	amputation wound swab
13.	*Enterobacter cloacae* 3354	ear
14.	*Enterobacter cloacae* 30612	ear
15.	*Enterobacter cloacae (ESBL+)* 30300	fistula
16.	*Enterobacter hormaechei* 30165	discharge from the prostate gland
17.	*Enterobacter hormaechei* 30426	urine
18.	*Enterobacter hormaechei* 30550	urine
19.	*Enterobacter hormaechei (ESBL+)* 29753	pus
20.	*Enterobacter kobei* 30367	sore

**Table 2:** Results of phage typing. CL—confluent lysis; SCL—semiconfluent lysis; +++—60 and more plaques; ++—20–60 plaques; OL—opaque lysis.

Bacterial Strain	Entb_43	Entb_45
*E. cloacae* 30345	CL	SCL
*E. cloacae* 29796	CL	CL
*E. cloacae* 3344	-	-
*E. cloacae* 3345	-	-
*E. cloacae* 30697	-	SCL
*E. cloacae* 30528	-	++
*E. cloacae* 30103	-	-
*E. cloacae* 29916	CL	SCL
*E. cloacae* 29779	SCL	SCL
*E. cloacae* 29731	OL	++
*E. cloacae* 30642	-	-
*E. cloacae* 30256	-	-
*E. cloacae* 3354	-	SCL
*E. cloacae* 30612	-	-
*E. cloacae* 30300	CL	SCL
*E. hormaechei* 30165	-	+++
*E. hormaechei* 30426	CL	SCL
*E. hormaechei* 30550	-	-
*E. hormaechei* 29753	OL	-
*E. kobei* 30367	-	++
*E. faecalis 3*	-	-
*E. faecalis 4*	-	-
*S. aureus 1*	-	-
*S. aureus 2*	-	-
*K. pneumoniae 7*	-	-
*A. baumannii 703*	-	-
*A. baumannii 3940*	-	-
*A. baumannii 1326*	-	-
*P. aeruginosa 5*	-	-
*P. aeruginosa PS735*	-	-
*E. coli 8*	-	-
*E. coli 9*	-	-

**Table 3:** Characterization of the newly isolated *Enterobacter*-specific bacteriophages.

Phage Symbol	Phage ICTV Name	Host Strain	Species Specificity	Source of Isolation	Lytic Spectrum
Entb_43	*Enterobacter* phage vB_EclM-43	*E. cloacae* 30345	*E. cloacae*, *E. hormaechei*	water sample from river (Thames, London, UK)—raw sample	8 of 20 strains (40%)
Entb_45	*Enterobacter* phage vB_EclM-45	*E. cloacae* 29796	*E. cloacae*, *E. hormaechei*, *E. kobei*	water sample from river (Fatima, Portugal)—raw sample	12 of 20 strains (60%)

**Table 4:** Dimensions of bacteriophages. Average measurements of ten virions are shown.

Phage Symbol	Family	Morphotype	Total Dimension [nm]	Capsid Length [nm]	Capsid Width [nm]	Capsid Diagonal [nm]	Tail Length [nm]	Tail Width [nm]	Base Plate Width [nm]
Entb_43	*Myoviridae*	A2	223.4	116.7	82.1	103.9	106.7	21.2	33
Entb_45	*Myoviridae*	A2	220.9	113.6	89.8	107.3	107.3	20.3	33.7

**Table 5:** Influence of different MOIs on the phage titers after amplification. The final phage titer results reflect the mean of the titers in at least triplicate.

Phage Name	MOI	Host Bacteria [CFU/mL]	Phage Titer [PFU/mL]	Phage Titer after Overnight Incubation [PFU/mL]
Entb_43	0.001	10^6^	10^3^	6.33 × 10^9^
	0.01	10^6^	10^4^	6.93 × 10^9^
	0.1	10^6^	10^5^	3.20 × 10^9^
	1	10^6^	10^6^	2.87 × 10^9^
	10	10^6^	10^7^	1.19 × 10^8^
	100	10^6^	10^8^	7.13 × 10^8^
	1000	10^6^	10^9^	8.80 × 10^8^
Entb_45	0.001	10^6^	10^3^	4.93 × 10^9^
	0.01	10^6^	10^4^	2.67 × 10^9^
	0.1	10^6^	10^5^	1.49 × 10^9^
	1	10^6^	10^6^	5.73 × 10^8^
	10	10^6^	10^7^	7.73 × 10^7^
	100	10^6^	10^8^	3.38 × 10^7^
	1000	10^6^	10^9^	5.53 × 10^7^

**Table 6:** Adsorption rate constant for phage Entb_43 and phage Entb_45.

Adsorption Rate Constant [mL/min]	
Entb_43	4.92 × 10^−10^
Entb_45	9.29 × 10^−10^

**Table 7:** Basic features of sequenced phages.

Phage Name	Genome Size [bp]	Start Codons [%]	Coverage	Genes	GC Content [%]
*Enterobacter* phage Entb_43	174 681	ATG: 95.3 GTG: 2.90 TTG: 1.81	902	positive strand: 240 negative strand: 41	39.7
*Enterobacter* phage Entb_45	172 771	ATG: 97.8 GTG: 1.48 TTG: 0.74	661	positive strand: 230 negative strand: 43	40

**Table 8:** Percentage of phages retaining activity after specified intervals of incubation with various disinfectants.

	Phage Entb_43				Phage Entb_45			
	5 min	10 min	20 min	30 min	5 min	10 min	20 min	30 min
10% dish soap	77.8 %	78.3 %	83.9 %	77.8 %	74.3 %	75.7 %	93.3 %	74.3 %
10% liquid soap	83.3 %	79.4 %	97.2 %	77.8 %	80.6 %	86.2 %	82.9 %	80 %
disinfectant (1) (diluted 2×)	65.3 %	41.4 %	17 %	7.8 %	15.6 %	2.1 %	0.1 %	0.01 %
10% hand wash gel	138.9 %	107 %	126.4 %	87.8 %	129.5 %	112 %	81.7 %	85.4 %
skin disinfectant (2) (diluted 2×)	120.6 %	130.6 %	87.5 %	67.1 %	61 %	80 %	68.6 %	80 %
70% ethanol	57.5 %	56.7 %	35.8 %	27 %	21 %	7.7 %	8.4 %	3 %

## Data Availability

The presented data are available on request from the corresponding author.

## Supplementary Materials

The following supporting information can be downloaded at: https://www.mdpi.com/article/10.3390/v14071518/s1, Figure S1. Kinetics of Entb_43 phage adsorption on *E. cloacae* 30345 and Entb_45 phage on *E. cloacae* 29796 at MOI = 0.1. Table S1. List of all genomes with over 70% similarity to *Enterobacter* phage Entb_43 genome. Table S2. List of all genomes with over 70% similarity to *Enterobacter* phage Entb_45 genome. Table S3. Similarity of each protein product of marker genes from *Enterobacter* phage Entb_43 to the closest relative classified by ICTV. Table S4. Similarity of each protein product of marker genes from *Enterobacter* phage Entb_45 to the closest relative classified by ICTV.
