# Supplementary material for: Two Newly Isolated Enterobacter-Specific Bacteriophages: Biological Properties and Stability Studies
Source: Viruses. 2022 Jul 12;14(7):1518. doi: 10.3390/v14071518 (PMC9319786; doi:10.3390/v14071518)
Supplement: Supplementary file 1 [file viruses-14-01518-s001.zip › viruses-1788978-supplementary.pdf]

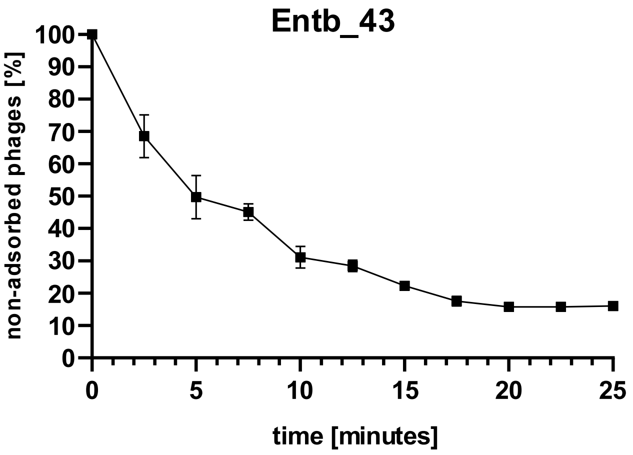

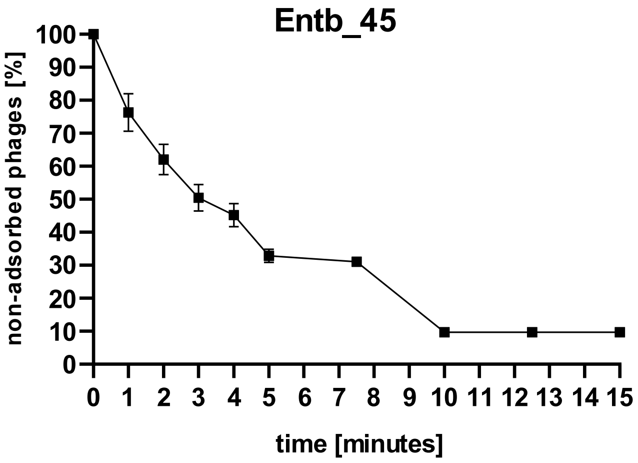

Figure S1. Kinetics of Entb_43 phage adsorption on *E. cloacae* 30345 and Entb_45 phage on *E. cloacae* 29796 at MOI=0.1. Error bars represent the standard deviation (± SD) of the mean phage titers.

Table S1. List of all genomes with over 70% similarity to *Enterobacter* phage Entb_43 genome. Query cover, e-value and identity determined by BLAST. Not classified – phages not officially classified by ICTV. Name, description and acc. no. as they appear in the GenBank database.

Description	Name	Query Cover	E-value	Identity	Acc. no.	Genus by ICTV
Klebsiella phage vB_KaeM_KaAlpha	*Klebsiella* phage vB_KaeM_KaAlpha	96%	0.0	96.55%	MN013084.1	not classified
Enterobacter phage PG7, complete genome	*Enterobacter* phage PG7	94%	0.0	96.37%	KJ101592.1	*Karamvirus*
Enterobacter phage vB-EclM_KMB19, complete genome	*Enterobacter* phage vB-EclM_KMB19	96%	0.0	97.48%	OL828290.1	not classified
Cronobacter phage Pet-CM3-4 genome assembly, chromosome: I	*Cronobacter* phage Pet-CM3-4	96%	0.0	97.77%	NC_055726.1	*Karamvirus*
Enterobacter phage vB-EclM_KMB17, complete genome	*Enterobacter* phage vB-EclM_KMB17	91%	0.0	92.25%	OL849997.1	not classified
Enterobacteria phage CC31, complete genome	*Enterobacter* phage CC31	90%	0.0	91.26%	GU323318.1	*Karamvirus*
Enterobacter phage vB-EclM_KMB20, complete genome	*Enterobacter* phage vB-EclM_KMB20	89%	0.0	90.81%	OL828291.1	not classified
Enterobacter phage myPSH1140, complete genome	*Enterobacter* phage myPSH1140	92%	0.0	90.06%	NC_055739.	*Karamvirus*

Table S2. List of all genomes with over 70% similarity to *Enterobacter* phage Entb_45 genome. Query cover, e-value and identity determined by BLAST. Not classified – phages not officially classified by ICTV. Name, description and acc. no. as they appear in the GenBank database.

Description	Name	Query Cover	E-value	Identity	Acc. no.	Genus by ICTV
Enterobacter phage ENC9, partial genome	*Enterobacter* phage ENC9	95%	0.0	97.24%	OL355124.1	not classified
Enterobacter phage vB_EclM_CIP9, complete genome	*Enterobacter* phage vB_EclM_CIP9	96%	0.0	98.28%	NC_048849.1	*Kanagawavirus*
Enterobacter phage vB_EhoM-IME523	*Enterobacter* phage vB_EhoM-IME523	91%	0.0	96.07%	MN087708.1	not classified
Enterobacter phage vB_EclM_Q7622, complete genome	*Enterobacter* phage vB_EclM_Q7622	90%	0.0	96.63%	OL989991.1	not classified
Kosakonia phage 305	*Kosakonia* phage 305	89%	0.0	94.54%	MZ348423.1	not classified
Edwardsiella phage PEi20 DNA, complete sequence	*Edwardsiella* phage PEi20	73%	0.0	81.17%	AP014714.1	*Kanagawavirus*
Edwardsiella phage PEi26 DNA, complete sequence	*Edwardsiella* phage PEi26	72%	0.0	81.17%	AP014715.1	*Kanagawavirus*

Table S3. Similarity of each protein product of marker genes from *Enterobacter* phage Entb_43 to the closest relative classified by ICTV. List of marker genes as recommended by ICTV for *Tevenvirinae* phages. .

Name	Protein Product	Max value to closest *Karamvirus* representative by ICTV		Name of a phage with most closely related marker protein.
		Pairwise identity [%]	Query cover [%]	
Gp14	Neck protein	98.9	100	*Cronobacter* phage Pet-CM3-4
Gp17	Terminase subunit	99.8	100	*Enterobacter* phage myPSH1140
Gp18	Tail sheath protein	98.6	100	*Enterobacter* phage PG7
Gp19	Tail tube protein	100	100	*Enterobacter* phage CC31
Gp22	Capsid assembly protein	99.6	100	*Enterobacter* phage CC31
Gp23	Major capsid protein	100	100	*Enterobacter* phage PG7
Gp41	Helicase	99.6	95.4	*Cronobacter* phage Pet-CM3-4
Gp44	Sliding clamp loader protein	100	100	*Cronobacter* phage Pet-CM3-4
Gp46	Exonuclease subunit	100	100	*Enterobacter* phage CC31
Gp55	Sigma factor	100	100	*Enterobacter* phage CC31
Gp47	Exonuclease subunit	100	100	all
Gp61	Primase	99.1	99.4	*Cronobacter* phage Pet-CM3-4
RegA	Translational regulatory protein	100	100	*Enterobacter* phage CC31
Uvsw	Helicase	99.4	100	*Enterobacter* phage myPSH1140

Table S4. Similarity of each protein product of marker genes from *Enterobacter* phage Entb_45 to the closest relative classified by ICTV. List of marker genes as recommended by ICTV for *Tevenvirinae* phages.

Name	Protein Product	Max value to closest Karamvirus representative by ICTV		Name of a phage with most closely related marker protein.
		Pairwise identity [%]	Query cover [%]	
Gp14	Neck protein	99.2	100	*Enterobacter* phage vB_EclM_CIP9
Gp17	Terminase subunit	99.5	100	*Enterobacter* phage vB_EclM_CIP9
Gp18	Tail sheath protein	99.8	100	*Enterobacter* phage vB_EclM_CIP9
Gp19	Tail tube protein	100	100	*Enterobacter* phage vB_EclM_CIP9
Gp22	Capsid assembly protein	100	100	*Enterobacter* phage vB_EclM_CIP9
Gp23	Major capsid protein	98.7	100	*Enterobacter* phage vB_EclM_CIP9
Gp41	Helicase	99.7	100	*Enterobacter* phage vB_EclM_CIP9
Gp44	Sliding clamp loader protein	91.2	97.6	*Edwardsiella* phage PEi20
Gp46	Exonuclease subunit	99.8	100	*Enterobacter* phage vB_EclM_CIP9
Gp55	Sigma factor	100	96.7	*Enterobacter* phage vB_EclM_CIP9
Gp47	Exonuclease subunit	99.1	100	*Enterobacter* phage vB_EclM_CIP9
Gp61	Primase	99.7	100	*Enterobacter* phage vB_EclM_CIP9
RegA	Translational regulatory protein	100	100	*Enterobacter* phage vB_EclM_CIP9
Uvsw	Helicase	99.8	100	*Enterobacter* phage vB_EclM_CIP9
